# The Interplay Between Sleep and Safety Outcomes in the Workplace: A Scoping Review and Bibliographic Analysis of the Literature

**DOI:** 10.3390/ijerph22040533

**Published:** 2025-03-31

**Authors:** Janet Mayowa Nwaogu, Albert P. C. Chan, John A. Naslund, Shahnawaz Anwer

**Affiliations:** 1 School of Property, Construction and Project Management, Royal Melbourne Institute of Technology University, GPO Box 2476, Melbourne, VIC 3001, Australia; 2 Department of Building and Real Estate, The Hong Kong Polytechnic University, Block Z, 181 Chatham Road South, Hung Hom, Hong Kong, China; albert.chan@polyu.edu.hk (A.P.C.C.); shah-nawaz.anwer@polyu.edu.hk (S.A.); 3 Department of Global Health and Social Medicine, Harvard Medical School, 641 Huntington Ave, Boston, MA 02115, USA; john_naslund@hms.harvard.edu

**Keywords:** sleep, safety, workplace incident, accident, injury

## Abstract

Occupational incidents comprising injuries and accidents remain a serious concern globally. With sleep deprivation and fatigue representing key drivers of many workplace incidents, one strategy to reduce occupational incidents is implementing effective sleep management systems. Yet, to date, there are complaints about the methodological approach in sleep–safety studies. The extent of work carried out with respect to the impact of sleep on safety outcomes needs to be reviewed to highlight the state of the art in the face of increasing technological advancement and changing lifestyle attitudes. A systematic search of the Scopus and PubMed databases retrieved 63 journal articles published up to 2023. The units of analysis included article performance and thematic analysis. It was deduced that workers in healthcare and construction have been the focus of most studies, pointing to the prevalence of safety issues in both these sectors. Most of the studies adopted a quantitative methodology employing validated sleep questionnaires, especially the Pittsburgh Sleep Quality Index. Using thematic analysis, the research focus was mapped into six areas, including sleep disorders, cognition and performance, and injury and accident prevention in the construction sector. In objective studies, alertness and cognitive performance were considered a proxy for sleep deprivation and safety performance. Harmonising sleep questionnaires is necessary to prevent excessive paperwork and ineffective safety systems. This study has the potential to provide occupational health and safety researchers outside of the medicine and psychology disciplines with knowledge on baseline information that could advance efforts to address sleep deprivation and the resulting safety concerns in the workplace.

## 1. Introduction

Employees across different sectors are subjected to varying stress levels, which can negatively impact their productivity, health, and safety. Murtin et al. [1] noted that about one-third of employees are strained at work, with about 10% being severely strained, although regional disparities exist. For instance, in Europe, 13–22% of the working population was reported to suffer job strain [2], while in Japan, more than half of the working population experienced job strain [3]. Such strain can cause excessive psychophysiological arousal, which can expose people to the risk of coronary heart disease and sleep disorders [4]. Following the demands of a day’s work, sleep is the only natural way to recover [5]. However, the ability to recuperate even when people sleep can be altered for several reasons, such as poor sleep quality and lifestyle behaviours [6]. This inability among the working population to recover appropriately from daily work stress has enormous consequences for both employers and employees. Sleep deprivation and sleep problems are becoming prevalent among the working population and pose significant health and safety risks to employees, with resultant cost implications for productivity and organisations [4,7].

Insufficient sleep has been associated with impaired cognitive functioning, more frequent errors, slower information processing, and diminished task performance [4]. This has severe safety implications, such as near misses, work-related injuries, and fatal accidents [8,9]. Likewise, the health impact of inadequate recovery could result in absenteeism, presenteeism, increased sick leave, and lower productivity [4]. In many settings, particularly in higher-income countries, organisations pay workers’ compensation for accidents or injury claims, lost work hours, and medical expenses [8,10]. With the possibility of attributing about 13% of work injuries to sleep problems [11], the role of sleep as a risk factor in accident prevention should not be overlooked and requires further investigation and debate. From a workplace safety perspective, ensuring good sleep quality among the workforce benefits employees, organisations, and society [12].

Research shows that workers who achieve quality sleep are less likely to experience workplace accidents or injuries, as they are more likely to exhibit safe behaviours [8,10]. Despite this, workplace safety has mainly focused on safety climate, which comprises awareness of risks and hazards, discussion of safety issues and safety-specific leadership training [8]. Brossoit et al. [8] suggest broadening the approaches to protect employees at work, such as considering interventions to foster healthy employee sleep habits, as employees who achieve quality sleep are more likely to demonstrate safe behaviours while at work and have a reduced risk of workplace accidents or injuries. Likewise, Fietze et al. [4] hinted that even when considering sleep as a part of safety, company health management has focused mainly on sleep problems in shift work. Sleep-related issues (such as absenteeism, work errors, and sleeping at work) and sleep disorders (such as insomnia, sleep apnea, restless legs syndrome, narcolepsy, and circadian rhythm sleep disorders) are shared across various occupations, whether they involve standard or nonstandard work hours. Sleep-related problems significantly affect workers’ health, waking function, and short-term and long-term wellbeing [13].

Over the years, research has increasingly recognised sleep duration, sleep quality, and sleep problems as potential risk factors for workplace injuries and accidents. Still, the complexities of the sleep–safety relationship are yet to be adequately understood. This is evident as researchers have been perturbed by the inadequate methodologies and epidemiological approaches within the sleep–safety literature, as well as limited attention across a diverse range of industrial sectors and work environments [8,10,14]. Kao et al. [10] observed that although the connection between sleep problems and workplace safety has been acknowledged, significant gaps remain in epidemiological approaches within the current sleep–safety literature. Similarly, Brossoit et al. [8] scrutinised the methodologies used in the workplace safety literature, highlighting a prevalent dependence on correlational or quasi-experimental designs instead of true experimental designs.

A review of state-of-the-art research on sleep–safety could highlight progress and suggest research directions. This calls for a review of the existing studies to point out the state of the art and highlight future directions, especially in the face of increasing technological advancements and changing lifestyle attitudes. Reviews on sleep research within the working population have been conducted, with most focusing on the cost implications of sleep, e.g., [7]. Others looked into the impact of sleep disorders such as sleep apnea [15], the impact of sleep-related problems in shift workers [16,17,18], and the sources of fatigue and sleepiness [19]. Using a systematic review approach, Glick et al. [7] examined the economic impact of insufficient and disturbed sleep among adult employee populations. Similarly, Wong et al. [20] conducted a scoping review to assess the economic impacts of nonstandard work hours and the related risk mitigation strategies. However, while both reviews addressed sleep-related factors, neither explored their relationship with workplace safety concerns. In contrast, Garbarino et al. [15] conducted a systematic review and meta-analysis examining the relationship between a particular sleep problem (i.e., obstructive sleep apnea) and workplace accidents among workers. Some other reviews have focused on specific work schedules or professions. For example, Alfonsi et al. [16] summarised the literature on sleep problems and their effects on night shift nurses and examined the psychosocial factors influencing the impacts, whereas Bauerle et al. [19] provided an overview of sleep- and fatigue-related issues in mining populations. These reviews either focused on specific worker groups, addressed particular sleep disorders, or did not explore the potential impact of sleep on workplace safety.

The present study moves the review studies forward by conducting a bibliographic and scoping review of the existing literature on sleep and safety among the working population over the last few decades, up until 2023. Unlike previous reviews, it is not limited to a specific occupation, sleep disorder, or work schedule. Scoping reviews involve systematically identifying and synthesising the literature in a field [21]. In contrast, a bibliographic review aids the quantitative analysis and visualisation of the research pattern and trends using bibliographic data, thereby improving insights into a research area [22]. The integration of both methods enhances the depth of analysis, offering qualitative insights through thematic mapping and quantitative insights through trends and impact metrics. This study is intended to synthesise the knowledge on the role sleep plays in the field of health and safety among the workforce. Another focus of this study is to provide health and safety researchers in occupations outside of the medicine and psychology disciplines with knowledge on baseline information that could advance their sleep and safety research efforts and inform the development and implementation of new practices aimed at responding to sleep concerns and their resulting detrimental consequences across different sectors.

Based on the preceding factors, this study aims to examine the attention paid to the role of sleep in workplace health to provide information on the prevalence of poor sleep, sleep problems, and their impact on having a safe workplace by using a scoping review and a bibliographic analysis of the literature. To achieve this aim, the following specific research questions are addressed in this study:

(a) How did research on sleep–safety among the working population evolve over the years up to 2023 regarding publications and citations?
  This question would help us to understand how research related to sleep–safety has expanded over the years, e.g., trends and working population research. It would identify and distinguish foundational work from emerging trends.
(b) Which journals have garnered the most impact?
  Research on sleep–safety spans multiple disciplines, making it essential to identify high-impact journals as key sources of influential findings. This helps to pinpoint publications driving innovation and policy and shaping both advancements in the field.
(c) What topics (keywords and themes) are associated with this research field?
  Research on sleep–safety among the working population spans several disciplines, including occupational health, psychology, medicine, and ergonomics. Identifying keywords and themes helps to categorise knowledge, providing a clearer picture of the scope. This would highlight how sleep–safety is conceptualised and studied across different work environments.

This study will enhance the existing body of knowledge by highlighting key articles for reference and current and emerging research areas within the field, informing practitioners and researchers of the latest advancements in sleep–safety research, and suggesting future research directions based on the identified gaps in the reviewed literature. In this study, the term “incident” follows Gharibi et al. [23]’s definition. It refers to unplanned events at work in terms of injury that interrupt task completion, including near misses and accidents.

## 2. Materials and Methods

This study combined a scoping review and bibliographic analysis to eliminate bias that might arise in the keywords and the development of themes. Scoping reviews are valuable for investigating the design and conduct of a research field [24]. A scoping review uses a systematic and iterative approach to identify and synthesise the literature in a particular field [21]. Bibliography (bibliographic analysis) is a quantitative approach to analysing the published scientific literature and research outputs. It involves measuring and evaluating patterns in publications, citations, and other scholarly data to objectively assess trends, impacts, and the developments in a particular field [22]. The bibliographic review was used to quantify, map, and visualise research output in the field of the study [22]. Thereafter, the articles were subjected to a scoping review of contents. This study employed Biblioshiny (version 4.3.0), the Shiny-based interface of the Bibliometrix R package, to conduct the bibliographic review. Biblioshiny is an open-source R tool (version 4.3.2, 31 October 2023) for science mapping based on the biblometrix package [25]. It was employed in this study because of its ability to perform comprehensive analysis, visual representation, and map thematic evolution in a research field.

Unlike systematic reviews and meta-analyses that need to be registered on public registries such as PROSPERO, it is not compulsory to register scoping reviews, but rather recommended [26]. Since PROSPERO does not accept scoping reviews, literature reviews, or mapping reviews [27], the protocol for this review was made available on the Open Science Framework at https://doi.org/10.17605/OSF.IO/6VXH2. This review study adopts the PRISMA framework and its extension for the scoping review (PRISMA-ScR) by Tricco et al. [28], which allows for systematically scrutinising research in a particular field.

### 2.1. Unit of Analysis Employed for Bibliographic Analysis

The units of analysis employed for the bibliographic analysis were (i) document citation analysis, (ii) journal analysis, and (iii) keywords (topic) trends analysis through thematic mapping and evolution.

#### 2.1.1. Document Citation Analysis

The document citation analysis was measured using the most locally and globally cited documents. The distinctions are important for analysing the relative influence of documents within a focused research field (local) versus their broader scientific impact (global). Most locally cited documents help to identify influential papers within a research field, focusing on internal citations among the documents under analysis. This implies that local citations come from other documents in the same dataset [25]. The most globally cited documents refer to those that have received the highest number of citations in the broader scientific literature outside the dataset. Based on how frequently they are cited in scientific databases like PubMed or Scopus, this analysis helps to identify documents that have made significant contributions to a field.

#### 2.1.2. Thematic Map

In bibliometric analysis, a thematic map is used to visualise research themes or topics based on their importance and development over time. It can identify the structure of a research field and highlight emerging or well-established topics. Two top-performing community algorithms (Walktrap and Louvain) were employed for community detection [29]. The Walktrap algorithm is stable, unlike Louvain, which is not stable (non-deterministic), and yields varied results with each run [29]. Although Louvain is unstable and would require two or more runs to be implemented, the Louvain algorithm was utilised to create the thematic map of the articles, due to its capability to generate a hierarchical or “multi-level” organisation of communities. This feature is essential for identifying the various levels of taxonomies present in psychological assessment instruments [29].

In bibliometrics, a thematic map comprises four quadrants, each denoting a distinct theme category: motor themes, niche themes, emerging or declining themes, and basic themes. Themes in the upper right quadrant are classified as motor themes, and those in the upper left quadrant are termed niche themes. In the lower left quadrant are basic themes, while in the lower right quadrant are emerging or disappearing themes. Motor themes are well-developed and essential for structuring a research field [30,31]. They are considered pivotal for developing and organising a research field, as they are externally linked to concepts pertinent to other closely related themes within the field [31].

Motor themes exhibit dense connections, indicating their significance. Niche themes are highly specialised, but isolated, because while they have strong internal connections, they have minimal external relevance, rendering them of marginal importance to the field of study [31]. Emerging or disappearing themes have low density and centrality, representing emerging or fading concepts within a research field. Basic themes are transversal and general themes that provide foundational concepts across various aspects of a research field. Although basic themes hold importance for the research field, they lack substantial development [31].

#### 2.1.3. Journal Analysis Approach

Bradford’s law of scattering was used to analyse the performance of the journals with respect to the topic being investigated. In Bradford’s law, the journals are arranged from the most articles retrieved to the least, and divided into three zones (Zone 1, Zone 2, and Zone 3). Bradford’s law of scattering suggests that in any field, only a few document sources (journals) are very productive, a large number are moderate producers, while a larger number of journals have constantly diminishing productivity [32,33]. According to Bradford’s law, a few highly productive article outlets occupy the top three positions of all cited articles, known as Zone 1; these are the most frequently cited outlets in the field of study and likely of highest interest to researchers in that field [32]. Zone 2 consists of journals with an average number of citations. Those in the bottom third, known as Zone 3, are journals of marginal importance, as they are seldom cited [32].

### 2.2. Data Retrieval Process

Data for the review was gathered by conducting a systematic search of the scientific literature on the relationship between sleep and safety issues in work settings. Articles containing terms related to sleep and safety in the workplace were gathered from Scopus and PubMed, as they host the most extensive repositories of peer-reviewed journals in the occupational health field [34]. The article search was limited to empirical studies written in English and published by 2023. Also, the search was limited to journal articles, because conference papers and book chapters are often preliminary works later published in journals.

The final search string applied on Scopus was (TITLE-ABS-KEY (“sleep” AND “safety” AND “workplace”) OR TITLE-ABS-KEY (“sleep” AND “problems” AND “workplace”) AND NOT TITLE-ABS-KEY (“child” OR “ethnicity” OR “pregnancy” OR “family” OR “primary health care”)) AND (LIMIT-TO (DOCTYPE, “ar”)) AND (LIMIT-TO (LANGUAGE, “English”)) and it returned 463 journal articles. On PubMed, using the search string ((“sleep” AND “safety” AND “workplace”) NOT (“sleep” AND “problems” AND “workplace”)) NOT (“child” OR “ethnicity” OR “pregnancy” OR “family” OR “primary health care”) limited to English language returned a total of 222 full-text documents including 47 reviews, 10 systematic reviews, and 2 meta-analyses, which were later eliminated.

**Figure 1:**
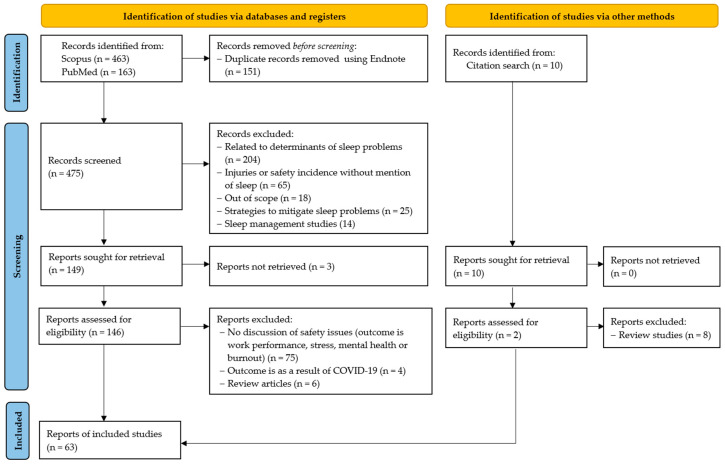
The PRISMA flow diagram for study selection.

The journal articles were exported to the Endnote reference manager, and duplicates were removed. The data retrieval and selection process followed the PRISMA guidelines, as shown in Figure 1. The inclusion criteria included journal articles that discussed or were associated with safety concerns, particularly injuries and accidents, as the primary outcome of sleep issues within the study population. Studies that associated cognitive performance and/or alertness from sleep issues with injuries/accidents were also considered. Upon reading the 146 full-text studies, sleep studies whose primary outcome was related to performance, stress, mental health, or burnout without safety concerns such as injuries, near misses, or accidents were excluded from the review.

After exclusion, 63 articles were retained for the review. The final database search and retrieval of articles took place in April 2024. Although the year of publication was set to any time up until 2023, the articles retained for this review were published between 2000 and 2023. As detailed in Table 1, the 63 articles had a total of 2835 references, 277 authors, and were published by 45 journals.

## 3. Results

**Figure 2:**
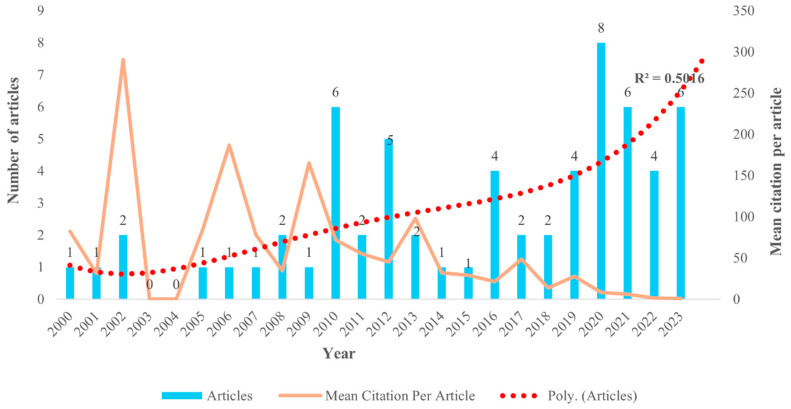
Publication rate and mean citations of documents per annum.

From 2000 to 2023, at least 63 documents related to the impact of sleep on safety at work were published in the peer-reviewed academic literature. As depicted in Figure 2, publications on the influence of sleep on occupational injury exhibited significant fluctuations between 2010 and 2023, with a notable acceleration in growth observed from 2020. A fourth-order polynomial trend line was applied to the publication year and the number of articles to forecast the publication rate. Although the trend line indicates a moderate fit, as it explains about 50.16% variability in the number of articles, it suggests an upward trend in the publication rate, especially in recent years, suggesting mounting interest and increased attention to this important topic area.

The studies focused on several industries: textiles, health, oil, rail, construction, education, retail, manufacturing, transportation, communication and technology, defence, agriculture, and services. Thus, the study population included textile factory workers, construction workers, healthcare professionals (nurses and physicians), psychologists, retail workers, teachers, drivers (taxi, truck, and train), police officers, and military personnel. The majority of the studies focused on nurses, followed by construction workers. Both professions are part of in-demand sectors; they are different yet similar in the challenges they are subjected to. Challenges common in healthcare and construction include physical demands, stress, high pressure, ageing workers, burnout, and labour shortages.

### 3.1. Sleep Assessment Tools Employed in the Studies

As detailed in Table 2, the research instruments used to assess sleep quantity, quality, and/or sleep issues included subjective and objective measures. The research instruments used to evaluate sleep objectively include polysomnography (e.g., Siesta Portable EEG system), the Pupillographic Sleepiness Test, and Actigraphy (SenseWear Armband, Actiwatch-2, Actiwatch Spectrum, and Actigraph GT9X Link accelerometers). Widely validated research instruments used to evaluate or diagnose sleep subjectively included the Pittsburgh Sleep Quality Index Scale (PSQI), Epworth Sleepiness Scale (ESS), Sleep Hygiene Index (SHI), STOP-BANG Questionnaire, Pittsburgh Sleep Diary, and Karolinska Sleep Questionnaire. These instruments have been validated among various populations and settings, yielding good reliability and internal consistency. Other questionnaires used to evaluate sleep include the Sleep Disorder Score Questionnaire (SDS-Q).

#### 3.1.1. Pittsburgh Sleep Quality Index Scale (PSQI)

The Pittsburgh Sleep Quality Index, developed by Buysse et al. [35], is a self-rated tool used to measure sleep quality to identify good and bad sleepers and disturbances among adults over a period of one month [35]. The PSQI is unique in that it combines quantitative and qualitative information. The PSQI contains 19 questions to be rated by the person under examination and 5 questions to be rated by the bed partner or roommate. The five questions are not tabulated in the PSQI scoring, as they are meant for clinical information. The 19 self-rated questions have seven components, namely (1) subjective sleep quality, (2) sleep latency, (3) sleep duration, (4) habitual sleep efficiency, (5) sleep disturbances, (6) use of sleep medication, and (7) daytime dysfunction owing to sleepiness.

Each domain is scored on a four-point scale ranging from 0 to 3, with 0 indicating no difficulty and 3 indicating severe difficulty [35,36]. The total score for the seven components ranges from 0 to 21, known as the global PSQI score [35]. As the score increases, the quality of sleep decreases. A global score above 5 (>5) indicates poor sleep quality, while a score of 5 or less (≤5) is considered good sleep quality [36]. The Pittsburgh Sleep Quality Index (PSQI) score can be used as an indicator of insomnia, as seen in Itani et al. [37].

#### 3.1.2. Epworth Sleepiness Scale (ESS)

The ESS was employed because of its ability to assess different sleep problems, particularly excessive daytime sleepiness and extremely excessive daytime sleepiness [37,38]. The ESS is an eight-item scale used to determine the level of daytime sleepiness. It is one of the most used scales to diagnose the likelihood of a person dozing off or falling asleep in different situations during the day [39]. The ESS is a four-point Likert scale rated from 0 to 3, resulting in a total score ranging from 0 to 24. A score above 10 in healthy adults indicates excessive daytime sleepiness [40]. The scoring categories are as follows: 0 to 10 = normal range, 11 to 14 = mild sleepiness, 15 to 17 = moderate sleepiness, and 18 or higher = severe sleepiness.

#### 3.1.3. Pittsburgh Sleep Diary

The Pittsburgh Sleep Diary (PghSD) is a well-regarded tool for tracking sleep patterns and behaviours over a period of two weeks. It has distinct sections to be filled out at bedtime and upon waking. The bedtime sections focus on events from the day leading up to sleep, while the waketime sections address the recently completed sleep period [41]. The PghSD provides a snapshot of a person’s sleep/wake behaviours [41], including entries on bedtime, wake time, sleep onset, awakenings, sleep quality, and daytime activities that might affect sleep. The diary provides detailed daily sleep insights, making it useful for personalised assessments. While it is used in research and clinical settings, it is not as widely validated as the Pittsburgh Sleep Quality Index (PSQI).

#### 3.1.4. Sleep Hygiene Index (SHI)

The Sleep Hygiene Index is a 13-item questionnaire used to diagnose sleep hygiene behaviour. Sleep hygiene encompasses the daily practices, habits, and environmental factors essential for enhancing nighttime sleep quality [42]. Sleep hygiene is defined as engaging in behaviours that facilitate sleep and avoiding those that interfere with it [43]. Each item of the SHI is rated on a five-point Likert scale ranging from 1 (never) to 5 (always), resulting in total scores from 0 to 65 [43], where 13 indicates no sleep hygiene issues, and 65 indicates significant sleep hygiene issues. However, some authors rate the SHI on a scale ranging from 0 (never) to 4 (always), with total scores ranging from 0 to 52 [42].

Higher scores on the SHI indicate poor sleep hygiene, as this shows that the person engages in more behaviours that are likely to compromise sleep hygiene [36]. According to [44], the SHI has an internal consistency of Cronbach alpha of 0.66 and good test–retest reliability of 0.71.

**Table 2:** The studies included in the study.

S/N	Article	Study Population	Research Method	Research Instrument for Sleep	Sleep Problem	The Outcome of Sleep Issue
1	Aderaw et al. [14]	Textile factory workers (n = 456)	Survey and interview	Questions for querying sleep disturbance problems are not specified	Sleep disturbance	Workplace injury
2	Alshareef [13]	Variety of occupations (n = 10,106)	Survey	Epworth Sleepiness Scale (ESS) Questions were used to query sleep quality, latency, and duration	Poor sleep quality Excessive daytime sleepiness (EDS)	Errors at work Falling asleep at work
3	Barnes and Wagner [45]	Miners (n = 14,301)	Interview	The number of minutes spent sleeping was used to deduce sleep quantity		Workplace injury
4	Booker et al. [36]	Healthcare shift workers (n = 406)	Survey	Pittsburgh Sleep Quality Index (PSQI) Sleep Hygiene Index (SHI)		Medical errors Workplace accidents Vehicular accidents
5	Brossoit et al. [46]	Construction workers (n = 222)	Survey	Pittsburgh Sleep Quality Index (PSQI) Sleep insufficiency measured using a single-item question from Buxton et al. [47]	Insomnia Fatigue	Cognitive failures Safety compliance and participation Minor injuries
6	Brossoit et al. [8]	Army and Air National Guard service members (n = 704)	Survey	Actigraph Pittsburgh Sleep Quality Index (PSQI) Patient-Reported Outcome Measurement Information System (PROMIS) Sleep Disturbance Scale	Insomnia Fatigue	Workplace safety behaviours Accidents and injuries
7	Buxton et al. [47]	Patient care workers (n = 1572)	Survey	Questions were used to query sleep duration, sleep insufficiency, and insomnia symptoms	Sleep deficiency	Pain Work interference Functional limitation
8	Çolak and Esin [48]	Nurses (n = 83)	Survey	Pittsburgh Sleep Quality Index (PSQI) Epworth Sleepiness Scale (ESS)	Daytime sleepiness	Psychomotor performance
9	Daley et al. [49]	University staff (n = 20)	Experiment and survey	Tobii Pro Eye Tracking Glasses Biopac bionomadix ECG	Sleep deprivation	Circadian process Cognitive performance
10	Das [50]	Brickfield workers, i.e., construction workers (n = 400)	Survey and interviews	The instrument used to measure sleep was not mentioned	Sleep disturbance	Increased risk of injury Reduced work performance Impact on health
11	Doss et al. [51]	Healthcare workers (n = 150)	Survey and interviews	Not mentioned	Sleep deprivation	Workplace hazard Reduced work performance Impact on health
12	Dutta [52]	Construction workers (n = 60)	Interviews and participant observations	Questions were used to query issues related to sleep and their effect	Fatigue	Increased risk of injury Reduced work productivity Physical and mental fatigue
13	Elfering et al. [53]	Software development and counselling (n = 40)	Experiment	Actigraphy SenseWear Armband		Workplace accident risk Cognitive failure
14	Fido and Ghali [54]	Blue collar workers in oil company (n = 200)	Survey	Pittsburgh Sleep Quality Index (PSQI)	Sleep disturbance Circadian disruption	Increased levels of errors Workplace accidents
15	Filtness and Naweed [55]	Train drivers (n = 28)	Focus group and experiment	Questions were used to inquire about the impact of fatigue and sleepiness	Sleepiness and fatigue Circadian disruption	Increased risk of spads (signals passed at danger) Cognitive impairment
16	Fisman et al. [56]	Healthcare workers (n = 350)	Survey and interview	Questions were used to deduce hours of sleep, hours at work, and vacation time	Fatigue Sleep deprivation	Workplace injury
17	Fletcher and Dawson [57]	Train drivers (n = 193)	Experiment and survey	Visual Analogue Scale Computerised OSPAT (Workplace Safety Performance Assessment Technology)	Fatigue Sleepiness Sleep deprivation	Alertness Cognitive and motor performance
18	Garbarino et al. [58]	Police officers (n = 218)	Survey and interview	Sleep Disorder Score Questionnaire (SDS-Q) Epworth Sleepiness Scale (ESS)	Insomnia Excessive daytime sleepiness Sleep apnea	Workplace injury and accident Near misses
19	Gharibi et al. [23]	Oil construction company workers (n = 661)	Survey	Epworth Sleepiness Scale (ESS) STOP-BANG Questionnaire	Excessive daytime sleepiness (EDS) Obstructive sleep apnea (OSA)	Workplace incidents
20	Itani et al. [37]	Vehicle factory workers (n = 714)	Survey	Pittsburgh Sleep Quality Index (PSQI) Epworth Sleepiness Scale (ESS)	Insomnia Daytime drowsiness (daytime disorders)	Workplace accident Near misses Fatigue, diminished memory and attention
21	Jay et al. [12]	Firefighters (n = 25)	Experiment and Survey	Polysomnography Siesta Portable EEG system Oxygen Desaturation Index (ODI) Subjective Sleep Quality Rating (instrument not mentioned)	Obstructive sleep apnea (OSA) Fatigue	Neurobehavioural performance impairment Safety concerns
22	Kao et al. [10]	Construction services company workers (n = 3510)	Survey	Jenkins Sleep Questionnaire	Insomnia	Safety behaviour Workplace accident and injury Reduced work productivity
23	Kessler et al. [59]	Not specified (n = 4991)	Survey	America Insomnia Survey (AIS)	Insomnia	Workplace and nonworkplace injury Reduced work productivity
24	Kling et al. [60]	Variety of occupations (n = 69,584)	Survey	Canadian Community Health Survey (CCHS) Cycle 1.1 2000–2001		Workplace injury
25	Kottwitz et al. [61]	Printing company employees (n = 27)	Experiment and survey	Actigraphy BodyMedia SenseWear Armband Sleep diary		Impaired cognitive functions, e.g., concentration issues
26	Lee et al. [62]	Variety of occupations (n = 26,468)	Survey	Questions used to inquire about fatigue and sleep	Sleep disturbance Fatigue	Workplace injury
27	Léger et al. [63]	Not specified (n = 631)	Survey	Assessed insomnia based on DSM-IV criteria	Insomnia	Workplace accident Concentration issue Increased absenteeism
28	Léger et al. [64]	Not specified (n = 738)	Survey	Pittsburgh Sleep Quality Index Scale (PSQI) Spiegel Sleep Inventory (SSI)	Insomnia	Absenteeism Workplace accident
29	Lin et al. [65]	Firefighters (emergency medical service workers) (n = 399)	Survey	Epworth Sleepiness Scale (ESS)	Daytime sleepiness Sleep deprivation	Workplace injury
30	Lindholm et al. [66]	Home care personnel (n = 665)	Survey	Karolinska Sleep Questionnaire	Sleep disturbance	Workplace injury
31	Linton and Bryngelsson [67]	Not specified (n = 2066)	Survey	Basic Nordic Sleep Questionnaire Uppsala Sleep Inventory	Insomnia Nighttime and early awakening	Workplace accident Concentration issue
32	Lombardi et al. [68]	Not specified (n = 101,891)	Survey	One question used to inquire about sleep		
33	Magnavita et al. [69]	Variety of occupations (n = 754)	Survey	Pittsburgh Sleep Quality Index (PSQI)		Fall in workers Syncope Presyncope
34	McMahon et al. [70]	Not specified (n = 23)	Experiment and survey	Karolinska Sleep Questionnaire Karolinska Drowsiness Test (KDT) Electroencephalogram (EEG) Actigraphy Actiwatch Spectrum (Philips Respironics)	Sleep deprivation	Drowsiness Attention
35	Melamed and Oksenberg [39]	Variety of occupations (n = 740)	Experiment and survey	Epworth Sleepiness Scale (ESS) Mini Sleep Questionnaire (MSQ)	Excessive daytime sleepiness	Workplace injury
36	Mohd et al. [71]	Electronic company workers (n = 255)	Survey	Karolinska Sleep Questionnaire	Daytime sleepiness	Workplace injury
37	Morassaei and Smith [72]	Variety of occupations *	Survey	Not mentioned	Sleep disturbance	Workplace injury
38	Muller et al. [73]	Minerals extraction and processing production workers (n = 48)	Survey	Pittsburgh Sleep Diary	Fatigue Sleepiness	Fatigue Drowsiness
39	Mulrine et al. [74]	Not specified (n = 24)	Experiment and survey	Polysomnography Karolinska Sleepiness Scale	Sleep deprivation Sleepiness	Alertness Cognitive performance
40	Mulugeta et al. [75]	Textile factory workers (n = 311)	Survey	Questions used to inquire about sleep		Workplace injury
41	Mutifasari and Ramdhan [76]	Truck drivers (n = 45)	Experiment and survey	Pittsburgh Sleep Quality Index (PSQI) Actigraph Fitbit	Sleep deprivation	Cognitive performance Workplace injury
42	Nakata [77]	Variety of occupations (n = 1891)	Survey	Questions used to inquire about sleep		Workplace injury
43	Nishimura et al. [78]	Nurses (n = 30)	Survey	Psychomotor vigilance task (PVT)	Sleep apnea syndrome (SAS)	Alertness Performance
44	Patterson et al. [79]	Emergency medical services (EMS) workers (n = 355)	Survey	Pittsburgh Sleep Quality Index (PSQI) Epworth Sleepiness Scale (ESS)	Daytime sleepiness Workplace fatigue	Fatigue Workplace injury
45	Petitta et al. [80]	Variety of occupations (n = 1000)	Survey	Karolinska Sleep Questionnaire	Difficulties falling asleep Sleep disturbance	Workplace accident and injury
46	Pirrallo et al. [38]	Emergency medical technicians (n = 1854)	Survey	The questions were adapted from the AAO-HNS Foundation SLEEP Study (Pre-Op) Enrolment Questionnaire and Epworth Sleepiness Scale (ESS)	Sleep-disordered breathing Risk of sleep apnea Excessive daytime sleepiness	Workplace accident and injury
47	Powell and Copping [5]	Construction workers ** (n = 100)	Experiment	Actigraph	Fatigue-related impairment Circadian rhythm disruption	Cognitive performance Increased accident risk
48	Powell and Copping [81]	Construction workers (n = 105)	Experiment	Actigraph	Sleep deprivation Fatigue-related impairment Circadian rhythm disruption	Accident risk Mental effectiveness Performance
49	Rashid et al. [82]	Medical doctors (n = 375)	Survey	Pittsburgh Sleep Quality Index (PSQI)	Sleep deprivation Fatigue	Cognitive functioning
50	Renn and Cote [83]	Not specified (n = 49)	Experiment and survey	Polysomnography Post-sleep questions	Sleep deprivation	Cognitive impacts
51	Robbins et al. [84]	Taxi drivers (n = 27)	Survey	Sleepiness was measured according to the following: Epworth Sleepiness Index (ESS) Insomnia Scale (AIS) The Berlin Questionnaire	Sleepiness Insomnia Obstructive sleep apnea	Workplace accident
52	Rosekind et al. [85]	Not specified (n = 4188)	Survey	Questions used to inquire about sleep	Insomnia Insufficient sleep syndrome (ISS)	Workability Nodding off while driving Workplace accidents
53	Sabbagh-Ehrlich et al. [86]	Truck drivers (n = 160)	Mixed methods (questionnaire and interview)	Pittsburgh Sleep Quality Index (PSQI)	Fatigue	Higher involvement in crashes with casualties
54	Sneddon et al. [87]	Drillers in oil and gas (n = 185)	Survey	Sleep Disruption Scale (Australian Maritime Safety Authority (AMSA))	Fatigue	Increased unsafe behaviour Accident involvement
55	Tait et al. [88]	Marine pilots (n = 35)	Experiment and survey	Actigraph GT9X Link accelerometers Sleep diary	Sleep disruption Fatigue	Cognitive performance Safety inferred
56	Thomas and Ferguson [89]	Flight crewmembers (n = 302 flight operations)	Survey	Subjective estimates of sleep		Operational performance Increased error rates
57	Uehli et al. [11]	Not specified (n = 731)	Survey	Pittsburgh Sleep Quality Index (PSQI) Sleep disorder was objectively diagnosed by a physician	Sleep disorder (no specific mentioned)	Workplace injury
58	Valent et al. [90]	Hospital workers (n = 200)	Survey	Epworth Sleepiness Scale Horne-Östberg Morningness–Eveningness Questionnaire (MEQ)	Sleep deprivation Fatigue	Workplace injury
59	Vargas-Garrido et al. [91]	Not specified (n = 1993)	Survey	Chilean Quality of Life Survey (ENCAVI) 2015–2016	Insomnia Daytime sleepiness	Commuting accident Workplace accident
60	Verma et al. [92]	Nurses (n = 480)	Survey	Sleep questionnaire	Sleep disturbance Fatigue	Workplace injury
61	Wilhelm et al. [93]	Construction workers (n = 34)	Experiment	Pupillographic Sleepiness Test (PST)	Daytime sleepiness	Risk of accident
62	Wilson et al. [94]	Nurses (n = 22)	Experiment and survey	Wrist Activity Monitor Actiwatch-2; Philips Respironics, Bend, Karolinska Sleepiness Scale (KSS)	Daytime sleepiness Fatigue	Performance (alertness) Safety risks inferred
63	Wong et al. [95]	Not specified Study 1 (n = 4238); study 2 (n = 202); study 3 (n = 71)	Survey	Karolinska Sleep Questionnaire Questions used to inquire about sleep difficulties and sleep quality		Workplace injury Cognitive failures

Note: * = number is in terms of lost time claims and non-lost time claims; ** = largely construction workers (83 are construction workers, 62 of which are engaged in field-based jobs); variety of occupations = occupations are specified, but the study population includes workers from multiple industries; and not specified = study includes working population, but specific occupations are not identified.

#### 3.1.5. STOP-Bang Questionnaire

The STOP-Bang Questionnaire developed by Chung et al. [96] is a validated screening tool for obstructive sleep apnea (OSA). It consists of eight questions, each coded “yes” or “no”, with each “yes” response scoring one point. STOP-Bang includes four subjective items (STOP: snoring, tiredness, observed apnea, and high blood pressure) and four demographic queries (Bang: BMI, age, neck circumference, and gender) [97]. The questionnaire is designed to be simple and easy to use, with cut-offs that help to determine the risk level for OSA. The maximum possible score on the STOP-Bang is 8 points, with 3 cut-offs where scoring 0 to 2 indicates a low risk of OSA, 3–4 is indicative of an intermediate risk of OSA, and 5–8 indicates a high risk of OSA [98]. Chung et al. [98] further explain that scoring 0 to 2 on the STOP-Bang questionnaire would mean the person is at low risk of OSA, and that the possibility of having moderate to severe sleep apnea can be confidently ruled out.

#### 3.1.6. Jenkins Sleep Problems Scale 

The Jenkins Sleep Problems Scale was developed by Jenkins et al. [99]. It is a four-item questionnaire used to measure sleep issues. It entails respondents rating how often they experienced specific sleep problems over the preceding four weeks using a six-point scale ranging from 0 (not at all) to 5 (almost every night, 22–28 days). The four items measure various aspects of sleep difficulties, including trouble falling asleep, waking up several times per night, trouble staying asleep, and waking up feeling tired [100]. The Jenkins Sleep Problems Scale measures three components of sleep problems: sleep onset, maintenance, and non-restorative sleep. The total score on the scale ranges from 0 (no sleep problems) to 20 (most sleep problems) [101], with higher scores indicating more frequent sleep problems. While there are no standardised cut-off points, some studies or clinical settings may use thresholds to identify clinically significant sleep problems. For instance, Monterrosa-Castro et al. [102] state that scoring 1 to 11 indicates low sleep disturbance, and that a score above 12 indicates a high frequency of sleep disturbance.

#### 3.1.7. Karolinska Sleep Questionnaire (KSQ)

The Karolinska Sleep Questionnaire, developed by researchers at the Karolinska Institute in Sweden, is a comprehensive self-report tool for measuring subjective sleep and sleepiness in various settings over a period of three months [66,103]. The KSQ has questions that relate to nocturnal sleep, sleep quality, snoring and cessation of breathing, sleepiness, and fatigue during the daytime [103]. The KSQ has been tested for reliability and validity in various populations, making it a trusted instrument in sleep research and occupational health studies. The KSQ does not have standardised cut-off points because it can also assess qualitative aspects of sleep. Instead, it provides a detailed profile of an individual’s sleep patterns, which is interpreted based on the specific context of a study.

#### 3.1.8. Karolinska Sleepiness Scale (KSS)

The Karolinska Sleepiness Scale (KSS) is a single-item scale that exists in two versions (a 9-point scale or a 10-point scale) used to measure subjective sleepiness at a given moment [104]. The scale has been used in various studies, including those related to attention and performance [105]. On the 9-point scale, 1 = extremely alert, 3 = alert, 7 = sleepy, but no effort to keep awake, 8 = sleepy and some effort to keep awake, and 9 = very sleepy, great effort to keep awake, fighting sleep. The modified version of KSS includes a 10th point, with 10 indicating “extremely sleepy, falls asleep all the time”. When using the KSS, if an individual self-assesses as 8 or 9, it signifies reduced alertness, and they should not perform safety-critical duties.

### 3.2. Document Citation Analysis for Sleep-Safety Research

#### 3.2.1. Most Locally Cited Documents

Most locally cited documents help to identify influential works within a research field by focusing on citations within the dataset under analysis. In other words, local citations refer to references from other documents included in the same collection [25]. The analysis of local citations provides insight into which studies have had the greatest influence within the specific body of literature on sleep and workplace safety. Among the 63 reviewed articles, only 37 local citations were recorded. Out of these, eight articles with at least two local citations accounted for about 70.3% of the total local citations accrued by the articles. This indicates that these articles serve as foundational or impactful studies within the field of sleep–safety research.

Details of the eight most locally cited documents are outlined in Table 3. Of the documents, Brossoit et al. [46] and Kling et al. [60] have the highest local citation count of five. Their prominence suggests that their findings strongly shaped subsequent research directions. Brossoit et al. [46] examined the impact of sleep on cognitive failures and safety in the workplace among construction workers. Similarly, Kling et al. [60] explored the relationship between sleep issues and work-related injuries among Canadian workers.

Other frequently cited studies, such as Barnes and Wagner [45], Nakata [77], and Powell and Copping [81], with four, three, and three local citations, respectively, further highlight the diverse angles through which the sleep–safety relationship has been investigated. For instance, Nakata [77] investigated the impact of extended work hours and inadequate sleep on workplace injuries, while Barnes and Wagner [45] introduced a unique temporal factor, “daylight saving time”, and its influence on sleep duration and injury rates. Using objective measures, Powell and Copping [81] examined sleep deprivation and its consequences for construction workers. They deduced that with inadequate sleep, the risk of accidents among construction workers increased by nine percent.

These findings highlight the growing recognition of the role sleep plays in occupational safety. The most locally cited documents tend to be those that either provide empirical evidence with strong methodological grounding or offer novel perspectives that have influenced subsequent research. The fact that only a few studies attract the majority of internal citations may suggest the need for better coordination and cross-referencing within the field.

#### 3.2.2. Most Globally Cited Documents

The most globally cited documents refer to articles that have received the highest number of citations in the broader scientific literature outside the dataset. The analysis of global citations offers insights into the wider influence and academic reach of the reviewed studies. Of the 63 articles analysed, 57 have received at least one global citation, totalling 2734 citations across the broader literature. Notably, 17 articles stand out with at least 50 global citations each, accounting for 76.2% (2083) of all global citations. This suggests that a relatively small number of studies have had a disproportionately large impact on shaping research related to sleep and workplace safety.

Table 4 details the 17 most globally cited documents. Regarding global citation, Léger et al. [63] entitled, “Medical and Socio-Professional Impact of Insomnia” has the highest global citation count of 386, highlighting its central role in advancing the understanding of the impact of insomnia. The fact that this article is cited at least 16 times annually indicates its sustained relevance in the field.

Similarly, Rosekind et al. [85] entitled “The cost of poor sleep: workplace productivity loss and associated costs”, has demonstrated exceptional influence, particularly in terms of citation velocity. Despite being published in 2010, it averages at least 17 citations per year, more than any other article in the dataset. Its focus on the economic burden of poor sleep, particularly through lost productivity, seems to have resonated widely across disciplines concerned with workforce performance and organisational costs. Sneddon et al. [87], with 166 citations and an annual average of 13, also ranks among the most influential, especially for its contributions to understanding the interplay between stress, fatigue, and situational awareness in the high-risk industry of offshore drilling environments, where sleep-related factors are critical to safety. Other topmost influential documents include Melamed and Oksenberg [39], Léger et al. [64], and Barnes and Wagner [45]. These findings underscore the multidisciplinary relevance of sleep research, especially when linked to occupational health, economic outcomes, and safety.

#### 3.2.3. Geographical Distribution of Study Population

The geographic distribution of the study population was deduced based on the location of the study populations. Table 5 summarises the countries where the study populations were located, providing insight into the global spread of sleep and workplace safety research. A total of 68 studies across 24 countries were included. The United States of America emerged as the most represented country, contributing 15 studies (22.1% of the total), indicating a strong research focus on this topic. This was followed by Australia with eight studies and Canada with six studies, reflecting similar interest in high-income nations.

European countries such as Italy (four studies), Switzerland, Sweden, France, Germany, and the United Kingdom also featured prominently, highlighting considerable research engagement on the subject across parts of Europe. Meanwhile, Asian countries or jurisdictions (including Japan, India, Malaysia, Israel, South Korea, Taiwan, Saudi Arabia, Singapore, and Iran) and Africa (particularly Ethiopia) were also represented. Notably, New Zealand contributed three studies, aligning with the trend observed in other developed nations.

Overall, the distribution indicates that the relationship between sleep and safety in the workplace has been examined across multiple regions. However, there appears to be a noticeable skew toward high-income and Western countries. This result is not surprising, as Western countries, particularly the USA, tend to be at the forefront of research and policy-making in several fields [106]. This geographic imbalance could suggest the need for more inclusive research that captures the diversity of work environments and sleep-related challenges faced by workers globally.

**Table 5:** Countries of study populations.

S/N	Country	Number of Studies
1	United States of America	15
2	Australia	8
3	Canada	6
4	Italy	4
5	Japan	3
6	India	3
7	Switzerland	3
8	New Zealand	3
9	United Kingdom	3
10	Malaysia	2
11	Israel	2
12	Sweden	2
13	France	2
14	Ethiopia	2
15	Iran	1
16	Saudi Arabia	1
17	South Korea	1
18	Taiwan	1
19	Kuwait	1
20	Singapore	1
21	Indonesia	1
22	Turkey	1
23	Chile	1
24	Germany	1
	**Total**	68

Note: The total number of studies exceeds 63 because some articles included participants from multiple countries (e.g., Wong et al. [107], United Kingdom, United States of America, and New Zealand; Fisman et al. [56], United States of America and Canada; Filtness and Naweed [55], Australia and New Zealand).

### 3.3. Representative Journals

It is necessary to highlight the journal outlets that researchers can visit for articles on this topic and to publish their works for increased visibility. Based on the articles retained for analysis, Bradford’s law of scattering revealed that the articles were published in 45 journal outlets, with 6 of the journal outlets in Zone 1 amassing 22 articles, 19 journals in Zone 2 accounting for 21 articles, and 20 journals having one article each in Zone 3 (see Table A1 in the Appendix A). As outlined in Table 6, Zone 1 journals, though fewer in number, accounted for the largest number of publications on the subject. The International Journal of Environmental Research and Public Health was the top journal source for publishing findings in the research field, followed by Sleep, Chronobiology International, the Journal of Workplace and Environmental Medicine, the Journal of Workplace Health Psychology, and the Journal of Sleep Research.

### 3.4. Keywords and Topic Trends Analysis

The keyword analysis consisted of a thematic map and thematic evolution.

#### 3.4.1. Thematic Analysis and Map for Sleep-Safety Research

A thematic map was employed to depict significant themes within the research domain. This visualisation aids in comprehending the research field by plotting its themes in a two-dimensional space [31]. The construction of the thematic map relied on the keywords index, which encompasses a vast array of terms, including author keywords, in contrast to the author’s keywords alone [108]. For each map, the volume of the spheres is proportional to the documents associated with each theme [31]. Also, the size of the circles indicates the thematic importance or frequency. The Walktrap community algorithm was utilised to achieve the keyword clusters. Upon using the Louvain algorithm, although similar themes to those in the Walktrap were derived, new words were revealed in the lower left quadrant. The Louvain analysis was run four times until a stable organisation of the keyword community was achieved.

**Figure 3:**
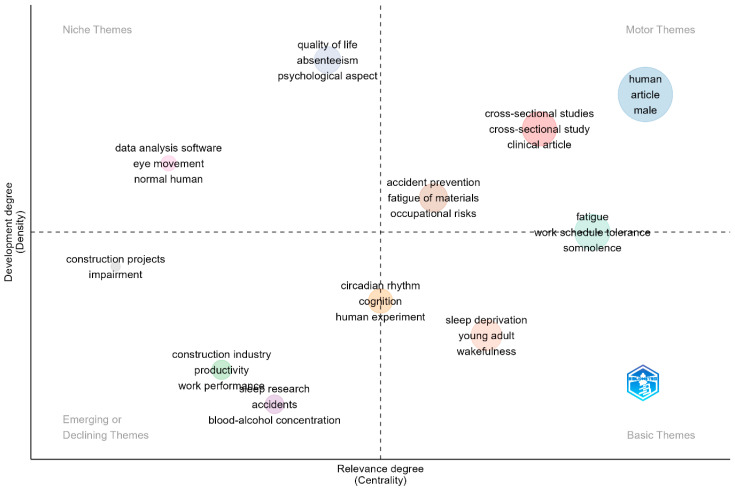
Thematic map of sleep and safety research using Walktrap.

**Figure 4:**
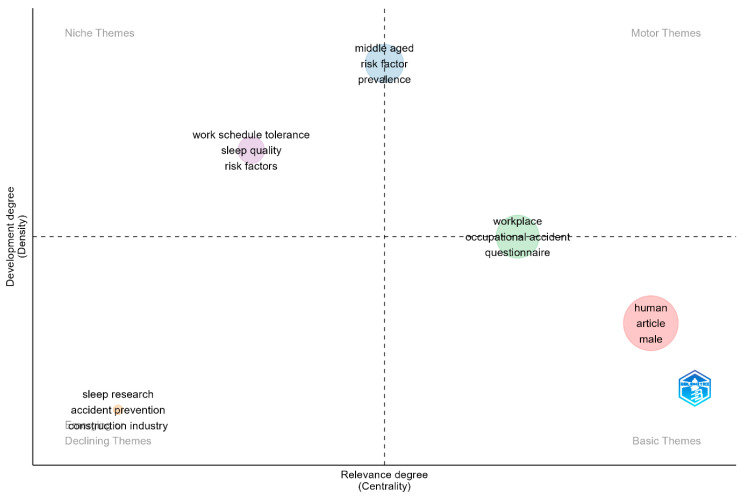
Thematic map of sleep and safety research using Louvain.

As indicated in Figure 3, the co-word analysis’ thematic map had ten clusters with Walktrap clustering. In the upper left quadrant are the niche themes, which are highly specialised themes, but isolated. The themes reflect the specialised and often distinctive areas in sleep–safety research. They are in two clusters related to quality of life (quality of life, absenteeism, and psychological aspect) and objective measures of sleep (data analysis software, eye movement, normal human). In the lower left quadrant are topics or areas gaining prominence, becoming increasingly relevant, or losing relevance within broader sleep–health research. In the left quadrant, the predominant themes comprise construction projects, impairment, construction industry, work performance, and productivity, suggesting a new interest or loss of focus on sleep research in construction. Likewise, in Figure 4, the themes in the lower left quadrant suggest emerging trends associated with sleep research in accident prevention within the construction industry.

In the upper right quadrant, motor themes are the most discussed topic in the field and essential for structuring any research in the field. In this case, the most discussed topics include fatigue and workplace risks. This quadrant also indicates that most studies were cross-sectional and clinical studies. In the lower right quadrant of Figure 3, sleep deprivation, young adults, and wakefulness represent essential discussion points in the studies. Both Figure 3 (upper right quadrant) and Figure 4 (the lower right quadrant) contain general themes such as human, young adult, adult, male, and female. These words represent broad themes associated with categorising written materials based on gender, such as considering the impact of gender on the relationship between sleep and safety. They represent central and foundational terms in sleep–safety studies. This aligns with Cobo et al. [31], who stated that themes in the lower right quadrant are transversal and general basic themes that provide foundational concepts across various aspects of a research field. In Figure 3, a cluster (fatigue, work schedule, and somnolence) appeared between the motor and basic themes and another (cognition, human experiment, and circadian rhythm) between the emerging/declining themes, suggesting that words have moderate significance within the field of study. It shows that they are developed and important, but not the primary focus.

#### 3.4.2. Thematic Evolution

**Figure 5:**
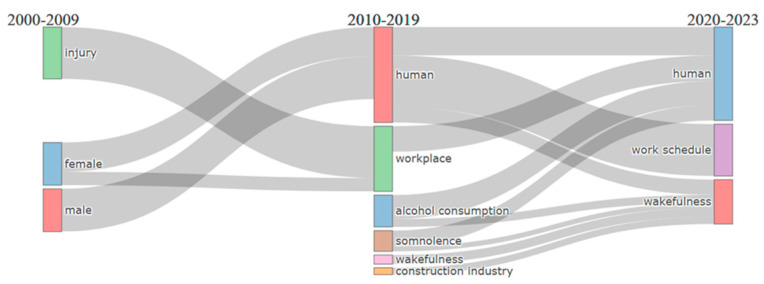
Thematic evolution of sleep and safety research using Walktrap.

The thematic evolution was performed on the keywords index following the default parameters. Figure 5 illustrates the progression of the research theme over time and its evolving focus. With two defined cutting points (2009 and 2019) aligned to changes in article production, it becomes evident that the research emphasis within the field has shifted gradually over the years. The coloured rectangular and square shapes represent the themes, while the thick grey lines connecting them signify the relationships between the keywords.

**Figure 6:**
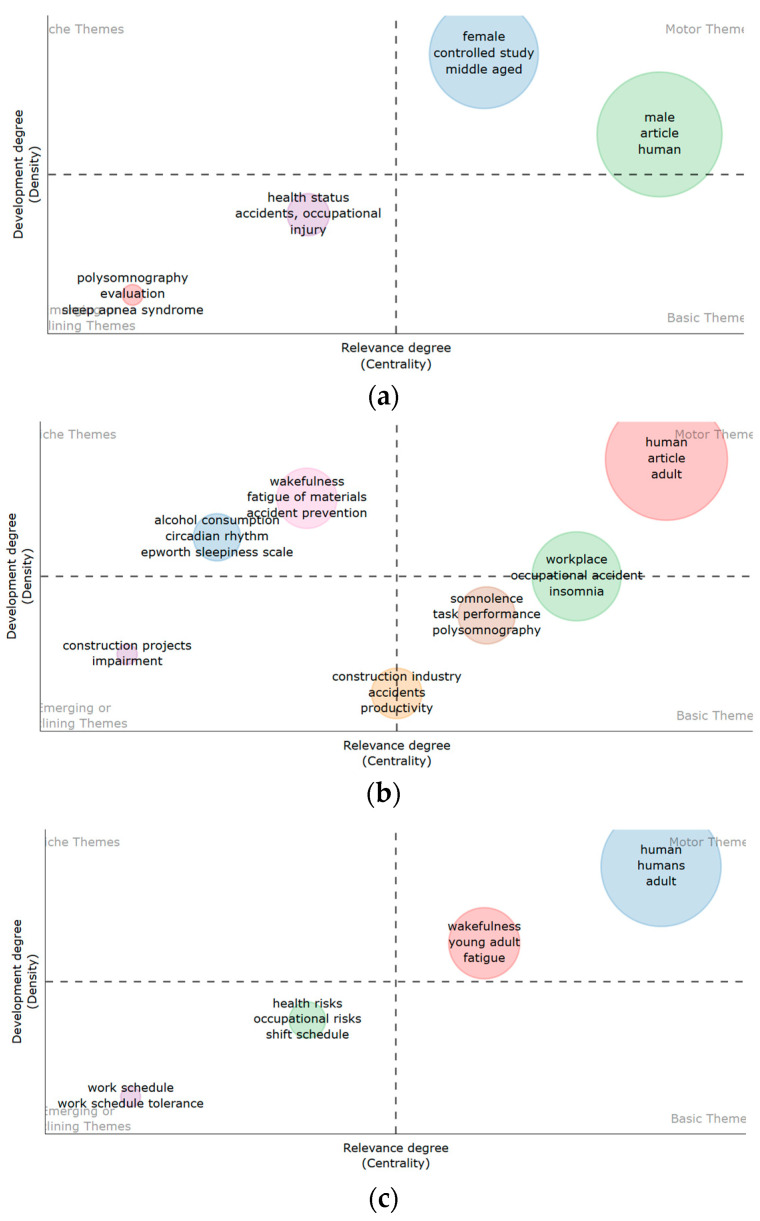
Thematic evolution and map of sleep and safety research from 2000 to 2023: (**a**) thematic map of articles published between 2000 and 2009; (**b**) thematic map of articles published between 2010 and 2019; and (**c**) thematic map of articles published between 2020 and 2023.

The analysis reveals that from 2000 to 2009, studies predominantly explored the topics of sleep and injury among specific groups. From 2010 to 2019, the focus expanded to include discussions on somnolence, wakefulness, and alcohol consumption in sleep studies, particularly within the construction industry. In the period spanning from 2020 to 2023, articles centred around attention and work schedule. As seen in Figure 6a, the term polysomnography was often used with sleep deprivation and circadian rhythm. Fatigue was often used with terms such as human, work schedule, attention, and insomnia (2020–2023). Workplace accidents were used with workers (2020–2023); specific terms among the studies included risk factors, workplace safety, work environment, and workload.

An in-depth analysis of the study focus within the three periods (2000–2009, 2010–2019, and 2020–2023) in the thematic evolution showed that motor themes across the years included, female, controlled study, adult, article, workplace, workplace accident, and insomnia. The terms point to the study focus, study design, and demographic of the research population. This is consistent with what formulates motor themes, as they consist of the most discussed topics in the field, which are essential for structuring any research field. The emerging themes from 2000 to 2009 included polysomnography, evaluation, and sleep apnea syndrome, pointing to the use of wearable technologies such as polysomnography devices to evaluate sleep and the possibility of sleep problems affecting worker attention on the job (see Figure 6a).

As seen in Figure 6b, emerging or declining themes from 2010 to 2019 consisted of one cluster and an intersection cluster: construction industry, accidents, and productivity. The study reveals that research on sleep and safety among construction workers emerged as an emerging theme between 2010 and 2019. However, a review of the articles signals that research focused on construction is still within the emerging zone. For instance, Gharibi et al. [23] opined that there is a dearth of research investigating the effects of sleep disorders on workplace and traffic accidents in the safety-critical construction sector.

In Figure 6c, two clusters make up the emerging/declining themes from 2020 onwards. “Work schedule and work schedule tolerance” are lower in the left quadrant and appear to still be less relevant and less developed, suggesting that they are either emerging or declining areas of focus within sleep studies related to shift work. In the same quadrant but upper, the themes “health risks, workplace risks, and shift schedule” appear to have moderate relevance and development, indicating they are important, but not the primary focus within shift work.

## 4. Discussion

Among the study population under review, sleep problems were not recorded in some studies. Even where specific sleep problems were not investigated, poor sleep duration and sleep quality were recorded. This suggests that inadequate sleep is prevalent across various disciplines. The population in the studies cut across a variety of industries. Although some studies focused on a particular industry, two sectors (health and construction) were mostly considered among those that considered a mix of study participants. The bibliographic analysis further revealed this, which mapped networks related to construction via the Walktrap and the Louvain algorithms. This could be because both health and construction are high-stress industries.

Fietze et al. [4] reported that the highest frequency of self-reported sleep problems came from service, health, and manufacturing employees, suggesting that irregular work schedules, such as shift work in the health and manufacturing industries, could be the reason. In this review, it is impossible to say which industry has the highest frequency of sleep problems or related issues because of the heterogeneous nature of the study participants. It could benefit our understanding if more studies on sleep and safety were conducted with a mix of study populations, especially focused on the nursing, construction, forestry, mining, oil, and service industries. This could aid comparability, more conclusive findings, and better interventions.

### 4.1. Areas of Sleep Incidence Research

Following the review of the articles using bibliographic means and systematic scrutiny of their content and research areas, six themes emerged, namely sleep disorders, cognition and performance, work schedule, impact on quality of life (physical health impact), injury and accident prevention in the construction sector, and demographic and lifestyle factors.

#### 4.1.1. Sleep Disorders

This theme captures sleep disorders as risk factors for accidents and injury prevalence among employees. Sleep disorders in the studies included sleep apnea [12,23], insomnia [8,59,64,109], and excessive daytime sleepiness [13,58,93]. These sleep disorders significantly impact quality of life, safety behaviour, and overall health. Employees with excessive daytime sleepiness (EDS) exhibited lower safety behaviour scores and more than often experienced workplace accidents and near misses [23]. It was found that all sleep problem variables in their study (difficulty in falling asleep, sleep interruptions, early awakenings, unsatisfactory sleep, short sleep duration (<7 h), daytime sleepiness, and habit of taking naps) were associated with injuries and accidents, as reflected in Garbarino et al. [58]. Among drivers, it was found that all the motor vehicle accidents reported were among drivers at high risk of obstructive sleep apnea [84].

In all the studies that looked into insomnia or insomnia symptoms, it was deduced that employees with insomnia were more likely than others to report workplace injuries, with elevated odd ratios [10,59,64]. Also, it was pointed out that insomnia increased absenteeism and dependence on medications for regulating physiological processes. By examining absenteeism, errors at work, accidents, and comorbidities between two good sleepers and insomniacs, Léger et al. [64] deduced that, unlike good sleepers, insomniacs tend to use medications for the central nervous system and systemic hormone preparations more frequently than people who do not have insomnia. Likewise, workers with insomnia were twice as likely to be absent from work or other obligations compared to those who sleep well.

Other impacts of these sleep problems in the literature include poor workability and poor mental health. In Lian et al. [110], insomniacs who slept for less than 5 h, 5–6 h, or 6–7 h were 3 times, 2 times, and 1.6 times, respectively, at risk of poor workability compared to those who slept more than 7 h and those who had normal sleeping patterns. Similarly, Omachi et al. [111], by evaluating work disability among workers with obstructive sleep apnea and excessive daytime sleepiness through two constructs, recent work disability and longer-term work duty modification, found that obstructive sleep apnea is associated with recent work disability, while the combination of obstructive sleep apnea and excessive daytime sleepiness significantly contributes to work disability and long-term work modification.

#### 4.1.2. Cognition and Performance

This theme includes alertness, absenteeism, circadian rhythm, eye tracking, and cognitive performance. Most of the studies in this category employed objective measures to examine sleep’s impact on workplace safety performance. Physiological metrics (heart rate variability, electrodermal activity, and eye tracking) are increasingly used to predict cognitive performance. Daley et al. [49], by examining whether aspects of prolonged wakefulness (sleep deprivation) can make a physiological metric a potent predictor, found that heart rate variability, electrodermal activity, and eye tracking provide insights into changes in cognitive performance and physiological states as a function of time awake and circadian rhythms. In Tait et al. [88], maritime pilots experienced reduced sleep duration and quality when on-call and after a night shift. Tait et al. reported that the lack of restorative sleep increased the level of fatigue, which could impair cognitive function and mood and increase the risk of performance breakdowns and accidents. With cognitive tests significantly correlating with actigraph-monitored sleep, Powell and Copping [5] indicated that sleep deprivation among construction workers adversely affects cognitive abilities and results in performance decrements. The study found that sleep deprivation among construction workers leads to impairments similar to those caused by alcohol consumption. Consequently, reduced mental effectiveness from inadequate sleep was correlated with blood alcohol concentration (BAC) levels, showing an increased risk of accidents. For instance, certain sleep-deprived states were equivalent to a BAC of 0.05%, which is considered unsafe for operating vehicles in many regions. This highlights the significant safety risks associated with sleep deprivation.

Several metrics have been employed in objectively estimating cognition, remarkably, heart rate variability, eye-tracking, and electrodermal activity [49]. Daley et al. [49] deduced that prolonged wakefulness affects various physiological metrics, which can help predict cognitive performance. Specifically, 31 out of 78 metrics were significantly influenced by time awake. The metrics were clustered into archetypal patterns, some of which aligned well with the Sleep, Activity, Fatigue, and Task Effectiveness (SAFTE) model, indicating a relationship with circadian and wakefulness factors. Despite similar daily sleep durations, night shift nurses showed a steady decline in performance, attributed to the circadian process affecting fatigue [93].

#### 4.1.3. Work Schedule

This theme relates to shift work. Nishimura et al. [78] reported that nurses’ ability to self-monitor decreased when they worked a night shift. In Wilson et al. [94], nurses working 12 h night shifts were found to experience more significant cognitive difficulties and increased sleepiness by the end of their shifts compared to those on day shifts. This highlights safety concerns, especially regarding potential risks to patients and nurses, and suggests organisational strategies to mitigate fatigue, such as considering different shift lengths or sanctioned workplace napping. The regular sleep schedule is to sleep at night, so working at night and sleeping during the day is off that schedule, as light during the day distorts melatonin levels, thus impairing sleep.

Mohd et al. [71] indicated that marital status and time of shift significantly affected workers’ sleepiness levels. The study deduced that the number of sleepy workers increased as the shift progressed. Mohd et al. suggest that the length of the shift might affect daytime sleepiness. Although studies have found that shift work and the duration of shiftwork affect sleep quality, some have argued otherwise, claiming that sleep quality is a function of the time of day when the shift occurs. Fletcher and Dawson [57] suggested that the time of the day for the shift work should be considered, not just the length of the shift. Aside from considering the time of shift work, studies researching melatonin levels in shift work and the length of work or shift should be considered.

#### 4.1.4. Impact on Quality of Life (Physical Health Impact)

It was reported that those at risk of sleep issues such as obstructive sleep apnea (OSA) were more at risk of high cholesterol, diabetes, and hypertension [84]. A significant correlation was found between poor sleep (quantity and quality) and increased workplace stress among truck drivers [76]. Fietze et al. [4] record that health hazards such as physical strain, physical environmental stress, and ergonomic environmental stress were heightened among workers with poor sleep.

#### 4.1.5. Injury and Accident Prevention in the Construction Sector

The incidents reported included actual injuries such as falls, sprains, and strains, scratches/abrasions, cuts/lacerations, and vehicular accidents [23,50,59]. These studies aimed to address minor and fatal incident concerns related to sleepiness, insomnia, sleep initiation, and maintenance disorders in construction workplaces. A number of studies found that inadequate sleep among construction workers resulted in an increased risk of accidents. For instance, sleeping less than the recommended eight hours per night resulted in a 9% higher risk of accidents for construction workers [81]. Poor sleep could induce fatigue-related impairments, such as poor alertness, which would expose workers to accident risks. Among construction workers, fatigue-related impairment was associated with higher workplace accident risks, as shown by Powell and Copping [5].

Construction involves many hazards, so workers need to be alert to avoid safety incidents. Wilhelm et al. [93], using objective measures, particularly the pupillographic sleepiness test, deduced that more than 60% of construction workers showed extreme sleepiness after eight hours of work, which persisted during longer shifts. In the study of construction workers, Brossoit et al. [46] found that those with more insomnia symptoms experienced more frequent cognitive failures at work, leading to lower compliance with safety protocols and procedures and higher rates of minor injuries at work. Also, it was deduced that construction workers with more significant sleep insufficiency (i.e., lower sleep quality) reported lower safety compliance.

Kao et al. [10] found that insufficient sleep decreased safety behaviours among construction workers, increasing the likelihood of workplace injuries. With a one-unit increase in insomnia, the likelihood of sustaining injuries was seen to increase by 0.10, while safety behaviour decreased by 0.06 [10]. The study observed that the negative relationship between insomnia and safety behaviours was weaker among construction workers with supervisors who prioritise safety. This suggests that safety-oriented and safety-supportive leadership could moderate the effect of insufficient sleep on safety performance.

#### 4.1.6. Demographic and Lifestyle Factors

Although the studies reviewed focused on the impact of sleep on safety performance among the working population, some risk factors for sleep-related issues were reported. Demographic factors such as age, gender, marital status, and work experience were reported to influence injury, sleep quality, and/or sleep-related incidents in different ways [10,13,14,66,71,75]. Alshareef [13] reported that younger individuals were found to have a higher risk of sleep-related errors at work while being single, female, and younger, and having a lower body mass index was associated with injury. According to Mohd et al. [71], marital status and work experience significantly influenced workers’ sleep levels.

In contrast to Alshareef [13], where younger workers were more susceptible to poor sleep and injuries, Mohd et al. [71] suggested that it was prevalent among older workers, adding that it may be due to decreased melatonin concentration, which reduces with age. Aderaw et al. [14] found that male workers, a younger age, and having sleep disturbances increased workplace injury. According to Kao et al. [10], the number of injuries resulting from sleep problems was higher in women. Lifestyle attitudes such as alcohol consumption and staying up at night can exacerbate poor sleep and invariably cause sleep-related incidents. For example, in Das [50], excessive alcohol consumption was deduced as a risk factor for work-related injuries among brickfield workers. More studies highlighted the role of demographics and lifestyle on sleep or workplace incidents, but little is known about the role of demographics and lifestyle on the sleep–workplace incidents path. Similarly to other fields of study, the results on demographic impacts were inconclusive.

## 5. Future Directions

In this review, poor sleep quality owing to sleepiness and other sleep-related issues was linked to adverse events and compromised safety behaviour among workers across various industries. Despite sleep research being conducted for decades, there is always room for more studies. For instance, a number of studies involved a variety of occupations, but work environments and psychosocial factors differ across occupations. Thus, insights gained from each group of diverse occupations may not inform us on the problems and required interventions. Strategies to reduce adverse outcomes related to sleep among the working population should be tailored towards the specific workplace environment [13]. This is necessary to avoid safety clutter by generalisation [112]. This would necessitate interventions targeting specific occupations, work environments, and sectors. Interventions targeting employees in high-stress sectors (like healthcare, manufacturing, and construction) where sleep deprivation is prevalent are required. Conversely, interventions such as harmonising sleep questionnaires and developing sleep quality models adaptable to diverse occupations are needed. Some points for further research focus on the following.

### 5.1. Sleep Questionnaires

With regard to sleep evaluation, although some studies did not adopt validated instruments, the majority of the studies adopted validated sleep questionnaires. In studies related to health management, all studies need to use validated instruments to ensure accurate, consistent, and credible measurements that can be compared, replicated, and applied in real-world settings [35,113]. Some sleep questionnaires employed had global scores, while others did not. Harmonising validated sleep questionnaires might be necessary, especially given calls for sleep assessments in workplace health management and the need to self-monitor sleep quality.

Appleton and Theorell-Haglöw [114] opine that the plethora of sleep questionnaires for assessing various dimensions of sleep may have hindered the advancement of sleep epidemiology. By harmonising sleep questionnaires, even if they evaluate various dimensions of sleep, they would include a global score that can be compared across studies, enhancing generalisability in sleep research findings, ease of use, and the interpretation of the instruments in self-management and company health management. A good way to solve the issue of self-monitoring sleep, given the limitations posed by diverse questionnaires with no harmonised sleep score, would be to promote the use of wearable devices with a sleep score to objectively monitor sleep quality, as they have shown good validity in sleep monitoring among diverse populations [115]. However, the use of wearable devices may be limited due to cost, comfort, and accessibility, so the harmonisation of sleep questionnaires is required so that more workers can use them.

### 5.2. Sleep Quality Model

Some studies employed consumer wearable devices to collect sleep data across various populations [76,94]. The utility of wearables (e.g., wristbands, armbands, smartwatches, headbands, rings, and sensor clips) has increased significantly in recent years because of their ability to measure physiological and behavioural processes related to stress [116]. There have been calls to integrate them into work settings to collect sleep data [76,115]. These wearable devices can also provide information on slips, trips, and fall propensity when worn during the day. They can also be used to assess physiological responses that correlate with accident risk, as shown in the studies reviewed, e.g., [76]. While wearable devices hold such benefits, they can cause sleep anxiety and discomfort, mainly because they must be worn securely to sleep to collect data properly [117,118], which could impede their use. To mitigate these shortcomings and the time taken to fill out sleep questionnaires, a sleep score model that can be used in Excel or simple calculation channels to estimate sleep quality from total sleep duration should be developed. This would also offer a cheaper sleep and safety measurement option, especially in low-resource settings, and can be applied across different settings [6].

There have been attempts to develop simple models that can be applied to optimise sleep and alertness, e.g., [119]. Kim and Park [119] developed a model for grading sleep habit levels considering various sleep dimensions. Vital-Lopez et al. [120] developed mathematical models to predict sleep latency and sleep duration as decision aids to predict efficacious sleep periods. Further studies should consider large-scale studies aimed at arriving at a sleep quality, sleep problems, and safety algorithms based on sleep duration and number of wakes.

### 5.3. Safety Management Audit

Sleep and fatigue management is critical for maintaining safety, particularly in industries that require high alertness and physical performance, such as healthcare, transportation, construction, and manufacturing, but there is a need to audit company health and safety interventions. With discussions around the overabundance of safety procedures in an attempt to manage safety [121,122], safety clutter can arise if employees have to fill several health instruments in company health management [112].

As detailed in Table 2, the studies reviewed used a number of research instruments for collecting sleep and incidence data. These instruments include objective tools (such as sleep tracking devices) and validated questionnaires (e.g., the Epworth Sleepiness Scale or Pittsburgh Sleep Quality Index). They also advocate for using those instruments in company health management, but caution must be taken against over-reliance on tools that could increase complexity without improving outcomes. Hence, there is a need for formal audits of sleep management systems to ensure that they are effective and adequately integrated into broader safety practices. These audits could focus on (i) the effectiveness of the current procedures in helping to reduce sleep-related incidents, (ii) how well sleep management protocols are integrated into the overall health and safety framework, and (iii) the practicality of sleep-related interventions so that they are not overly burdensome or confusing for employees.

### 5.4. Methodological Gaps

A number of the studies reviewed called for methodological rigour in sleep incidence research. It is essential to address existing methodological gaps in sleep incidence research to enhance the reliability of findings. Sometimes, when casual relationships between sleep and safety issues are expected, it is usually not deduced, especially when the incidence rates are not adequately documented or recorded. For instance, although Robbins et al. [84] found that all drivers who reported being involved in an accident also had a high risk of obstructive sleep apnea, the relationship was insignificant. This is not because poor sleep is not a risk factor of heightened incident occurrence, but it may be because the number of incidents is small or due to recall issues. There is a high risk of bias, especially given that many organisations do not have a reporting system for occupational incidents and sleep. Sometimes, safety outcomes are measured using documented objective data, but Gharibi et al. [23] opined that with respect to safety performance, that is not an effective method, because occupational incidents are frequently not recorded because of fear of punishment from management.

Further studies on sleep–safety issues should explore mixed methods involving experimental designs, documented objective data, and subjective tools to better understand the relationships between sleep and safety issues, particularly regarding workplace incidents. This would also allow for some triangulation of data. Information on work conditions or lifestyle behaviours that enhance sleep–safety problems can be missed if data are collected only objectively [23]. Thus, collecting data using a multifaceted approach provides additional information for each collection method. This is imperative to mitigate existing methodological problems and improve the quality of findings.

### 5.5. Effect of Sleep Issue Intensity

Fletcher and Dawson [57] mentioned that experimental studies on the level of employee alertness in the workplace are required because the chances of near misses, injuries, and accidents increase when cognitive alertness reduces. Since then, several studies have considered using experimental means. Although the pathway between sleep, alertness, and incidence occurrence has been established, the gap still needs to be adequately considered. In particular, the intensity and ability of sleep problems to cause reduced alertness invariably, cognitive performance and productivity may vary with the severity of sleep issues. Thus, there is a need to consider how alertness changes with the intensity of sleep issues. Takano et al. [123] deduced that moderate and severe insomnia severity had a significantly greater association with presenteeism.

## 6. Limitations

The scope of this study did not extend to the psychological impact of sleep deprivation. Instead, the study focused on the effects of sleep deprivation on safety outcomes, particularly incidents such as injuries, accidents, and near misses at work. Additionally, the articles retrieved may be limited based on the search string, languages of articles, and databases searched, which may affect the number of articles retained. However, to reduce the possibility of missing essential findings, studies that considered alertness and cognitive performance were considered a proxy for injury, accidents, and near misses. This is because decreased alertness can increase the likelihood of incidents in the workplace.

## 7. Conclusions

Studies have examined how sleep deprivation contributes to workplace accidents, injuries, and reduced productivity. This study conducted a bibliographic and scoping review of 63 peer-reviewed journal articles on sleep and safety outcomes among the working population to deduce how the studies evolved regarding publication citations over the years up until 2023 and topics associated with the research field. Regarding publications citations, two categories of performance (local citation and global citation) were established. It was deduced that among the articles analysed, Brossoit et al. [46], Kling et al. [60], Barnes and Wagner [45], Nakata [77], and Powell and Copping [5] were the most cited articles, otherwise known as locally cited articles. Léger et al. [63], Rosekind et al. [85], Melamed and Oksenberg [39], Léger et al. [64], and Sneddon et al. [87] were the most globally cited articles, which points to their relevance in this field of research. This study deduced that workers in the health and construction industry have been the focus of most studies on the impact of sleep on safety.

The majority of the studies adopted quantitative methodologies employing validated sleep questionnaires, including the Pittsburgh Sleep Quality Index (PSQI) to assess sleep quality, and the Epworth Sleepiness Scale (ESS) and Karolinska Sleepiness Scale (KSS) to assess sleepiness. Tools or instruments used to evaluate sleep objectively included actigraph (SenseWear Armband, Actiwatch-2, Actiwatch Spectrum, and Actigraph GT9X Link accelerometers), polysomnography devices (e.g., Siesta Portable EEG system), and consumer-grade activity trackers with sleep features (e.g., Fitbit). In terms of the geographical distribution of the study population, the USA, Australia, Canada, and Italy were the most represented countries among the included studies. There is a need for broader research on the impact of sleep on workplace safety that reflects the diversity of global work environments and the varying sleep-related challenges workers face worldwide.

The keyword analysis identified six research areas: sleep disorders, cognition and performance, work schedule, impact on quality of life (physical health impact), injury and accident prevention in the construction sector, and demographic and lifestyle factors. This study considered studies on the impact of sleep on safety outcomes, while future reviews may look into the effect of sleep on employees’ health, particularly those evaluating mental health and psychological wellbeing outcomes. In view of the increasing call for an overhaul of safety practices that do not amount to protecting workers themselves, particularly criticisms of paperwork, addressing sleep should be part of any health and safety management strategy for this workplace. Still, it should be integrated in such a way that sleep management does not add to filling in lengthy questionnaires. It was deduced that most recent studies that objectively evaluated the impact of sleep quality on safety outcomes in the workplace considered alertness and cognitive performance as a proxy for sleep deprivation and safety performance.

Following the analysis, this study suggests the need to harmonise validated sleep questionnaires for ease of use and to aid comparability of results across studies. It also advocates for using those instruments in company health management, but caution must be taken against over-reliance on tools that could increase complexity without improving outcomes. Future studies should focus on auditing sleep management systems to ensure that they are effective and adequately integrated into broader safety practices. This study highlights articles within the field of study, informing practitioners and researchers of advancements in sleep–safety research and suggesting future research directions.

## Figures and Tables

**Table 1:** Summary of the data retrieved.

Category	Description	Results
Main Information about Data		
	Timespan	2000:2023
	Sources (journals, books, etc.)	45
	Documents (articles)	63
	Annual growth rate for articles	8.1
	Average age per document	8.65
	Average citations per document (article)	43.4
	References	2835
Document Contents		
	Keywords plus (ID)	604
	Author’s keywords (DE)	215
Authors		
	Authors	277
	Authors of single-authored documents	4
Author Collaboration		
	Single-authored documents	4
	Co-authors per document	4.62
	International co-authorships, %	20.63
Document Type(s)		
	Journal articles	63

**Table 3:** Most locally cited documents.

S/N	Article	DOI	Study Population	Local Citations (LC)	Global Citations (GC)	LC/GC Ratio (%)
1	Brossoit et al. [46]	https://doi.org/10.1037/ocp0000139	Construction workers in the US	5	50	10
2	Kling et al. [60]	https://doi.org/10.1093/sleep/33.5.611	* Cross-section of the Canadian working population	5	70	7.41
3	Barnes and Wagner [45]	https://doi.org/10.1037/a0015320	Mining workers in the US	4	165	2.42
4	Nakata [77]	https://doi.org/10.1111/j.1365-2869.2011.00910.x	* Male employees in Tokyo	3	48	6.25
5	Powell and Copping [81]	https://doi.org/10.1061/(ASCE)CO.1943-7862.0000211	Construction workers in Canada	3	57	5.27
6	Wilson et al. [94]	https://doi.org/10.1016/j.aap.2017.09.023	Nurses in Saudi Arabia	2	52	3.85
7	Rosekind et al. [85]	https://doi.org/10.1097/JOM.0b013e3181c78c30	* Employees across different industries in the US	2	266	0.75
8	Kessler et al. [59]	https://doi.org/10.5665/sleep.1884	* Employees across different industries in the US	2	72	2.78

Note: * = the study population is not specific to any particular industry.

**Table 4:** Most globally impactful documents.

S/N	Article	Journal	DOI	Study Population	Total Citations (TC)	TC per Year
1	Léger et al. [63]	Sleep	https://doi.org/10.1093/sleep/25.6.621	* Cross-section of the French working population	386	16.78
2	Rosekind et al. [85]	Journal of Workplace and Environmental Medicine	https://doi.org/10.1097/JOM.0b013e3181c78c30	* Employees across different industries in the US	266	17.73
3	Melamed and Oksenberg [39]	Sleep	https://doi.org/10.1093/sleep/25.3.315	* Employees across different industrial plants in Israel	195	8.48
4	Léger et al. [64]	Sleep	https://doi.org/10.1093/sleep/29.2.171	* Cross-section of the French working population	187	9.84
5	Sneddon et al. [87]	Safety Science	https://doi.org/10.1016/j.ssci.2012.05.027	Drilling personnel in the UK	166	13.83
6	Barnes and Wagner [45]	J Appl Psychol	https://doi.org/10.1037/a0015320	Mining workers in the United States	165	10.31
7	Sabbagh-Ehrlich et al. [86]	Injury Prev	https://doi.org/10.1136/ip.2004.007682	Truck drivers in Israel	85	4.25
8	Linton and Bryngelsson [67]	J Occup Rehabil	https://doi.org/10.1023/A:1009408204694	* Employees across different industries in Sweden	82	3.28
9	Fisman et al. [56]	Infect Control Hosp Epidemiol	https://doi.org/10.1086/510569	* Employee healthcare workers across the United States and Canada	78	4.33
10	Kessler et al. [59]	Sleep	https://doi.org/10.5665/sleep.1884	* Employees across different industries in the US	72	5.54
11	Kling et al. [60]	Sleep	https://doi.org/10.1093/sleep/33.5.611	* Cross-section of the Canadian working population	70	4.67
12	Aderaw et al. [14]	J Trop Med	https://doi.org/10.1155/2011/657275	Textile factory workers in Ethiopia	62	4.43
13	Kao et al. [10]	J Occup Health Psychol	https://doi.org/10.1037/a0039144	Construction personnel	58	6.44
14	Powell and Copping [81]	Journal of Construction Engineering and Management	https://doi.org/10.1061/(ASCE)CO.1943-7862.0000211	Construction workers in Canada	57	3.8
15	Wilson et al. [94]	Accid Anal Prev	https://doi.org/10.1016/j.aap.2017.09.023	Nurses in Saudi Arabia	52	8.67
16	Filtness and Naweed [55]	Applied Ergonomics	https://doi.org/10.1016/j.apergo.2016.10.009	Train drivers in Australia and New Zealand	52	6.5
17	Brossoit et al. [46]	Journal of Workplace Health Psychology	https://doi.org/10.1037/ocp0000139	Construction personnel in the US	50	8.33

Note: * = the study population is not specific to any particular industry.

**Table 6:** Top journal outlets.

S/N	Sources (Journal Outlets)	Frequency	Zone	Articles per Zone
1	International Journal of Environmental Research and Public Health	5	Zone 1	22
2	Sleep	5	Zone 1	
3	Chronobiology International	3	Zone 1	
4	Journal of Workplace and Environmental Medicine	3	Zone 1	
5	Journal of Workplace Health Psychology	3	Zone 1	
6	Journal of Sleep Research	3	Zone 1	

## Data Availability

Some or all data and codes supporting this study’s findings are available from the corresponding author upon reasonable request.

## Appendix A

**Table A1:** Article sources.

S/N	Sources (Journal Outlets)	Frequency	Zone	Articles per Zone
1	International Journal of Environmental Research and Public Health	5	1	22
2	Sleep	5	1	
3	Chronobiology International	3	1	
4	Journal of Workplace and Environmental Medicine	3	1	
5	Journal of Workplace Health Psychology	3	1	
6	Journal of Sleep Research	3	1	
7	Nature and Science of Sleep	2	2	21
8	Sleep and Vigilance	2	2	
9	Accident Analysis and Prevention	1	2	
10	Annals of Workplace Hygiene	1	2	
11	Applied Ergonomics	1	2	
12	Australian Journal of Rural Health	1	2	
13	Aviation Space and Environmental Medicine	1	2	
14	Biomedical Signal Processing and Control	1	2	
15	BMC Public Health	1	2	
16	BMJ Open	1	2	
17	Current Psychology	1	2	
18	Ergonomics	1	2	
19	Infection Control and Hospital Epidemiology	1	2	
20	Injury Prevention	1	2	
21	International Journal of Experimental Research and Review	1	2	
22	International Journal of Industrial Ergonomics	1	2	
23	International Journal of Workplace and Environmental Medicine	1	2	
24	International Journal of Workplace Medicine and Environmental Health	1	2	
25	International Journal of Psychophysiology	1	2	
26	International Nursing Review	1	3	20
27	Iranian Journal of Nursing and Midwifery Research	1	3	
28	Journal of Applied Psychology	1	3	
29	Journal of Construction Engineering and Management	1	3	
30	Journal of Engineering, Design and Technology	1	3	
31	Journal of Workplace Rehabilitation	1	3	
32	Journal of Tropical Medicine	1	3	
33	Malaysian Journal of Medicine and Health Sciences	1	3	
34	Medical Principles and Practice	1	3	
35	Nordic Journal of Working Life Studies	1	3	
36	Workplace and Environmental Medicine	1	3	
37	PLoS ONE	1	3	
38	Prehospital Emergency Care	1	3	
39	Safety Science	1	3	
40	Sleep and Biological Rhythms	1	3	
41	Sleep and Breathing	1	3	
42	Sleep Medicine	1	3	
43	Somnology	1	3	
44	Transportation Research Part F: Traffic Psychology and Behaviour	1	3	
45	Work and Stress	1	3	
